# What constitutes “social complexity” and “social intelligence” in birds? Lessons from ravens

**DOI:** 10.1007/s00265-018-2607-2

**Published:** 2019-01-19

**Authors:** Palmyre H. Boucherie, Matthias-Claudio Loretto, Jorg J. M. Massen, Thomas Bugnyar

**Affiliations:** 1 Department of Cognitive Biology, University of Vienna, Vienna, Austria; 2 Konrad Lorenz Forschungsstelle, Core Facility for Behaviour and Cognition, University of Vienna, Vienna, Austria

**Keywords:** Social complexity, Social cognition, Corvids, Monogamy, Non-breeding period

## Abstract

In the last decades, the assumption that complex social life is cognitively challenging, and thus can drive mental evolution, has received much support from empirical studies in nonhuman primates. While extending the scope to other mammals and birds, different views have been adopted on what constitutes social complexity and which specific cognitive skills are selected for. Notably, many avian species form “open” groups as non-breeders (i.e., seasonally and before sexual maturity) that have been largely ignored as potential sources of social complexity. Reviewing 30 years of research on ravens, we illustrate the socio-ecological conditions faced by these birds as non-breeders and discuss how these relate to their socio-cognitive skills. We argue that the non-breeding period is key to understand raven social life and, to a larger extent, avian social life in general. We furthermore emphasize how the combination of the large-scale perspective (defining social system components: e.g., social organization, mating system) and the individual-scale perspective on social systems allows to better capture the complete set of social challenges experienced by individuals throughout their life, ultimately resulting on a more comprehensive understanding of species’ social complexity.

## Introduction

### Proxies for social complexity and “social intelligence”

While it is widely acknowledged that animal societies differ in social complexity, there has been little consensus about what is exactly meant by this term (Freeberg et al. 2012; Bradbury and Vehrencamp 2014; Bergman and Beehner, 2015; Rubenstein et al. 2016; Kappeler 2019, topical collection on Social complexity). In vertebrates, notably primates, social complexity has long been viewed through the prism of social cognition (Jolly 1966; Whiten and Byrne 1988; Barrett et al. 2007; Byrne and Bates 2007; but see in insects: Sheehan and Tibbetts 2008; Lihoreau et al. 2012). The “social intelligence hypothesis” assumes that the challenges posed in coping with the variability and unpredictability of the social environment have been a major evolutionary force for the evolution of cognition (Jolly 1966; Humphrey 1976; Byrne and Whiten 1988). While all animals face daily challenges in their physical environment, group-living animals additionally have to adapt their behavior and decisions to that of conspecifics (Kummer et al. 1974; Whiten and Byrne 1988; Bergman et al. 2003). Various proxies arose from the need to quantify and compare social complexity and “social intelligence” across species. In particular, the social brain hypothesis suggested that dealing with an increasing number of conspecifics might go along with a qualitative and quantitative improvement of information processing abilities and, possibly, larger brains (Dunbar 1992, 1998; but see González-Forero and Gardner 2018). As a consequence, brain size (neocortex ratio) and group size were the first and most widely used measures of cognitive abilities and social complexity, respectively (e.g., Sawaguchi 1992; Dunbar 1995; Reader and Laland 2002; Burish et al. 2004; Ashton et al. 2018).

So far, the social brain hypothesis has received strong support in haplorrhines primates (Dunbar 1992; but see DeCasien et al. 2017; Powell et al. 2017). However, the relationship between sociality and brain size is less clear in other taxa (Barton 1996; Shultz and Dunbar 2006; MacLean et al. 2009), and particularly unclear in birds (Beauchamp and Fernandez-Juricic 2004; Burish et al. 2004; Iwaniuk and Arnold 2004; Iwaniuk and Hurd 2005). Part of the explanation for this probably lies in the inadequacy of both brain and group size as proxies of cognitive and social complexity (Kappeler 2019, in topical collection on Social complexity). Specifically, with regard to social complexity, it can be challenging to identify the size of fundamental social units, especially when species tend to form “open” groups, characterized by high degrees of fission-fusion dynamics (Aureli et al. 2008). In addition, group size does not allow to differentiate a flock of a hundred fish from a troop of a hundred baboons. In both cases, we may see complex motion patterns that can be explained by simple rules of attraction and repulsion (Couzin et al. 2002; Sumpter 2006; Farine et al. 2017); yet, baboons live in multilevel societies characterized by the interweaving of multiple layers of social units, themselves based on the formation of individualized relationships (Kummer 1968). Hence, compared to fish shoals, baboon troops are considered to be socially more complex and cognitively demanding.

### Social relationships

Moving beyond group size, it was then suggested that not only the quantity of partners matters, but also the type and quality of the relationship that binds individuals (Cords and Aureli 2000; Dunbar and Shultz 2007; Emery et al. 2007; Shultz and Dunbar 2007, 2010). Along this line, the number of differentiated relationships that individuals have in a group has been proposed as a better proxy to quantify social complexity (Freeberg et al. 2012; Bergman and Beehner 2015). Using this definition, the number and diversity of partners which individuals regularly encounter and interact with, the nature of their interactions, as well as the context in which they occur, are at the core of social complexity (Freeberg, 2012; Bergman and Beehner, 2015; Fischer et al. 2017). Relationships are by definition inferred from the nature, frequency, and patterns of repeated interactions occurring among group members (Hinde 1976), while the group structure is inferred from the network of all relationships emerging in the group (Hinde 1976). The emergence and maintenance of relationships requires at least individual recognition and the ability to keep track of social interactions (Massen et al. 2010; Dunbar 2018a). Dealing with an increasing number of relationships thus implies an increase in information processing capacities and, possibly, larger brains (Dunbar 1992, 1998). In addition, individuals may profit from inferring relationships between others, which may also go along with a qualitative improvement of processing abilities (Whiten and Byrne 1988; Bergman et al. 2003; Bond et al. 2003; Call and Tomasello 2008).

### Social system components

Several intrinsic group constraints can mediate the nature and patterning of social interactions, and thus ultimately, the degree of social complexity of the systems (Krause and Ruxton 2002; Lehmann et al. 2016; Dunbar 2018b; Kappeler 2019, topical collection on Social complexity). Each social system results from the combination of its social organization, mating and care system, and its social structure, and each change in one component is likely to affect the others (Kappeler and van Schaik 2002). Furthermore, social systems are not static; instead, they can be remarkably flexible (Lott 1991; Henzi et al. 2009; Streatfeild et al. 2011; Schradin 2013). Within the same species, populations can exhibit different social systems according to the combination of external (e.g., ecological) and internal pressures (e.g. competition for food or reproduction) they experience (e.g., Baglione et al. 2002a, b; Schradin et al. 2010). Substantial intraspecific variations in social organization (i.e., group size and composition) can also be observed in various species according to seasons, breeding activity, and changes in ecological factors (e.g., food availability, predation risk). In birds, the pair and by extension the family unit is often the fundamental social-unit of most systems (i.e., reproductive partners and yearly juveniles, except for colonial or cooperative breeders). While this is true during the breeding season, birds often join larger flocks for foraging or roosting outside the breeding season, or when food resources are scarce (Develey and Peres 2000; South and Pruett-Jones 2000; Amano et al. 2006; de Moura et al. 2010; see also Silk et al. 2014). It is essential to consider such intraspecific variations in social systems if we want to characterize adequately their degree of social complexity, in particular in birds (see also Ashton et al. 2018).

### Taking an individual perspective on social systems: The effect of age and life history stages on individual sociality

Even though characterizing the different components of a system (i.e., organization, mating and care system, structure) provides a valuable framework to evaluate its potential complexity as a whole, it is not necessarily an accurate representation of what individuals experience on the day-to-day basis (Aureli and Schino 2019, topical collection on Social complexity). Over a lifetime, we can expect substantial variation in the social environments individuals are exposed to, notably in species with a long life span. Individuals’ life histories are paced by major events such as sexual maturity or the first breeding attempt, that are also likely to affect the set of partners they might seek to associate with. In numerous bird species, non-breeder individuals—immature juveniles and ‘floaters’ (i.e., sexually mature individuals that are not reproducing)—tend to flock together (e.g., Henderson and Hart 1991; Braun et al. 2012), which likely maximizes their survival before/outside of breeding (Powell 1974; Wright et al. 2003). In long-term monogamous species like corvids, juveniles tend to affiliate with multiple partners, of both same and/or opposite sex, and often preferentially with siblings; over time, they interact more and more exclusively with a single opposite sex partner, eventually resulting in a pair bond (de Kort et al. 2006; von Bayern et al. 2007; Scheid et al. 2008; Loretto et al. 2012).

A characteristic feature of the non-breeding period in birds is the high variability and unpredictability of the social environment, typically going along with dispersal and frequent joining/leaving of group members (i.e., high degree of fission-fusion dynamic; Silk et al. 2014). Thus, this period seems to reflect an increased diversity in social opportunities and challenges, which supports the argument that the complexity of avian social systems cannot be captured from the sole perspective of adults’—breeders—social system. However, to date, we have relatively few data on the diversity and dynamics of social relationships that may emerge outside of the family unit in avian species, and in particular outside of the breeding context (i.e., before the first reproductive attempt and outside of the breeding season).

### Towards a combined approach of (avian) social complexity

In the subsequent sections, we will use the common raven (*Corvus corax*) as an example to illustrate how a combination of top-down and bottom-up approaches (i.e., characterization of social systems’ components; individual perspective on social challenges) may allow us to capture the diversity of social environments experienced by individuals across a lifetime, and thus better apprehend the species’ social complexity. By reviewing the research of the last 30 years (which started in the late 1980s with the seminal work of Bernd Heinrich and got momentum in the last 10 years by studies from our own group), we will outline (i) which socio-ecological conditions ravens face as non-breeders, and (ii) how this relates to their social behavior and socio-cognitive skills.

## Raven social life

**Fig. 1:**
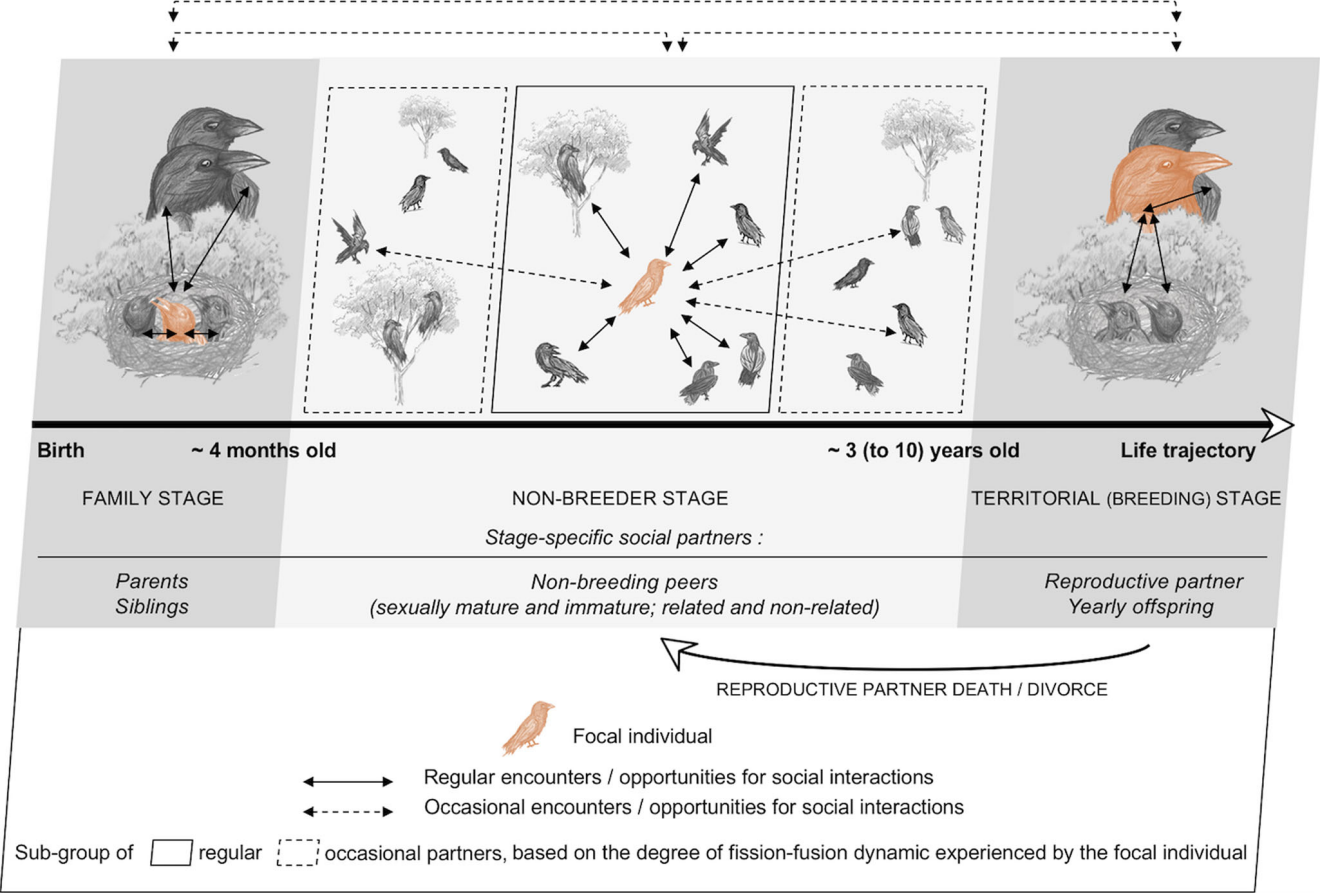
Schematic representation of the variations in the set of stage-specific social partners, across ravens’ three fundamental life stages (i.e., family stage, non-breeder stage, territorial stage). Note that in case of divorce or death of the reproductive partner, individuals return to the non-breeder stage. All stage-specific partners are listed below the figure. The bird in orange represents a theoretical focal individual going through all three life stages. Figure and drawings by Palmyre H. Boucherie

Common ravens are long-term monogamous and breeding pairs defend a territory of approximately 10 km^2^ year-round (Haffer and Kirchner 1993; Rösner and Selva 2005). According to their breeding system, ravens’ social life could thus be characterized as “moderately complex” (Boarman and Heinrich 1999), as the quality and prevalence of the pair bond may constrain the formation of social relationships with other conspecifics. Breeding is a life stage which ravens reach earliest at an age of 3–4 years (Ratcliffe 1997; Webb et al. 2009), but it can also take up to 10 years and more (unpublished data from our field site). With a life expectancy in the wild of 10–15 years (occasionally also 20–30 years; Haffer and Kirchner 1993; Fransson et al. 2010), ravens do spend a significant part of their life in the non-breeder state (Fig. 1). Characterizing the social complexity solely based on their breeding system may thus be misleading.

Non-breeding ravens tend to form temporary flocks that are composed of juvenile, immature birds but also of adults that do not have a partner and/or a breeding territory (Heinrich 1989; Braun and Bugnyar 2012; Loretto et al. 2016b). Flocks vary in size: they are largest for roosting (up to > 1000 birds; Engel et al. 1992; Wright et al. 2003), likely because of predator protection, and relatively small for socializing (< 10 birds; Braun and Bugnyar 2012). During foraging, ravens can aggregate in large numbers at food bonanzas like carcasses of large mammals or garbage dumps (> 100 birds; Boarman and Heinrich 1999), but mostly they forage in smaller groups (< 30 birds; Heinrich 1989; Dall and Wright 2009), using night roosts as information centers (Marzluff et al. 1996; Wright et al. 2003) and recruiting others via food-associated calls (Heinrich 1988). Being in a group increases the individuals’ chances of gaining access to food that is monopolized by territorial breeders or defended by predators (Marzluff and Heinrich 1991; Stahler et al. 2002); however, it also results in high food competition among non-breeders, expressed in physical aggression, kleptoparasitism, and the pilfering of food caches (Marzluff and Heinrich 1991; Heinrich and Pepper 1998; Bugnyar and Kotrschal 2002).

Taken together, non-breeding ravens’ social life can be characterized as flexible with regard to group size and composition and as a mix of cooperation and competition concerning the social opportunities and challenges faced (Fig. 1). Hence, this life stage features (i) a degree of social complexity, which is not represented by the breeding system, and (ii) some of the key factors discussed for the evolution of social cognition (e.g., competition; Machiavellian intelligence hypothesis: Byrne and Whiten 1988; and cooperation; Vygotskian intelligence hypothesis: Moll and Tomasello 2007). However, a minimalistic alternative view would be that raven groups represent anonymous crowds with individuals interacting on the basis of simple decision rules. The critical question is thus whether or not individual identities and their social relationships matter in those groups?

## Study population and group dynamics

Since 2007, our working group has been monitoring the population of wild ravens in the valley Almtal in the Northern Austrian Alps. On average, 50 ravens aggregate every day at a zoo, the Cumberland Wildpark Grünau (15 ravens in summer and up to 120 in winter; 47.80° N, 13.95° E), to scrounge food from captive animals such as wild boars (*Sus scrofa*), bears (*Ursus arctos*), and wolves (*Canis lupus*; Drack and Kotrschal 1995). Since the beginning of our long-term study, more than 300 ravens have been trapped and individually marked. During this procedure, we collect blood samples for the genetic analysis of sex and kinship and estimate the ravens’ age classes based on mouth and feather coloration (Heinrich 1994; Heinrich and Marzluff 1992). Presence-absence data and behavioral observations on these marked birds reveal that the groups in the park consist mainly of non-breeders (> 90%). The sex ratio of the non-breeders is even and the age classes are distributed in about 20% juveniles (ravens in their first year), 60% sub adults (between 1 and 3 years of age), and 20% adults (older than 3 years). In addition to the non-breeders, 7–12 breeding pairs of surrounding territories use the park opportunistically, i.e., during winter when food is scarce or during the period of raising their offspring (Loretto et al. 2017).

Contrary to breeders, non-breeders do not defend a territory and they can be highly vagrant (Heinrich et al. 1994). Using GPS tracking, we recently found a remarkable individual variation concerning the degree of vagrancy: some birds roam over thousands of square kilometers (km^2^), visiting many different food sources; others move between several food sources covering around 100 km^2^; and again, others rely on a single food source and can be found in an area of only a few km^2^ over months to years (Loretto et al. 2016a, 2017). These results match our long-term observations in the zoo, where we categorized non-breeders according to their presence/absence pattern as “rare visitors” (highly vagrant birds), “regular visitors” (birds using the food source in the study area from time to time), and “locals” (individuals that are observed almost daily in the park; Braun and Bugnyar 2012).

In general, the food sources used by ravens in the Eastern Alps are typically of anthropogenic origin (e.g., game parks, compost stations, and garbage dumps), corroborating the findings of other studies in Western Europe (Huber 1991; Wright et al. 2003; Loretto et al. 2016a) as well as in rural parts of North America (e.g., Boarman et al. 2006; Webb et al. 2012). Most of these anthropogenic food sources are highly predictable, “refilled” on a regular basis and used by many ravens at a time (often 30–50 individuals per day). Birds that are feeding at the same site usually aggregate at dusk at one or few night roosts in the surrounding of that site. Those birds that stay at a particular site across months (“locals”) have a very high probability of repeatedly meeting other ravens with similar presence patterns (locals meet locals on around 70% of the days) and a moderately high likelihood of meeting ravens that pass by at that site from time to time (the same “visitors” on around 40% of the days; Loretto et al. 2017). Moreover, GPS tracking revealed that even when ravens range over thousands of square kilometers; they can still be found in repeated associations with the same individuals at different foraging sites, located more than 100 km away from each other (Loretto et al. 2017).

The differentiated use of foraging sites leads to a high degree of fission-fusion dynamics operating on different spatio-temporal scales. First, per site and day, non-breeder groups split up into small units when departing from the night roost in the morning; they may form bigger units during foraging and smaller units during socializing and eventually gather at the same night roost in the evening again (Braun et al. 2012; Loretto et al. 2017). Second, across days, birds may stay at the site or move to another food source at another site and join the local non-breeder group there (Loretto et al. 2017). Similar spatio-temporal patterns have been documented in other studies on non-breeding ravens (Heinrich 1988, 1989; Dall and Wright 2009) and linked to their scavenging life style: i.e., when they exploit a temporary food source like a carcass, ravens show the daily pattern described above; upon depletion of the source, individuals seem to independently leave and join other groups (Heinrich et al. 1994). Our findings add two important points to this picture: (i) size and composition of non-breeder groups may change independently of the availability of food, i.e., some individuals come and go although the food supply is constant and (ii) non-breeder groups may develop structure, i.e., some individuals prefer to stay at a site and thus become “locals” that meet each other on a daily basis (Braun and Bugnyar 2012; Loretto et al. 2017). Which factors determine individuals’ degree of vagrancy is still unknown. What can be said, however, is that a “local” life style creates conditions that meet several criteria of promoting individualized relationships and socio-cognitive skills, i.e., meeting repeatedly, competing for the same resources (access to food, partners, territories), and competing for status (formation of dominance rank hierarchies).

## Raven social relationships and socio-cognitive skills

Ravens are renowned for using social information to find and exploit ephemeral food sources (Heinrich 1989, 2011). They can passively share information at their night roosts or actively recruit others to foraging sites (Marzluff et al. 1996). Specifically, when ravens face difficulties in accessing food, they may give food-associated calls (i.e., “yells” or “haa” calls) that attract nearby ravens (Heinrich and Marzluff 1991) and meet the criteria of functional reference (Heinrich and Marzluff 1991; Bugnyar et al. 2001). Given the dynamics of raven groups (see above), one could argue that food calls attract any raven close by, and consequently, lead to the formation of anonymous crowds (Heinrich 1989). However, recent studies found large inter-individual variation in terms of: (i) how ravens sound (Boeckle et al. 2012), (ii) how often they call (Sierro 2015), and (iii) whom they respond to (Szipl et al. 2015).

Parts of the differences in call structure and call rate can be explained by the birds’ age and sex, with adults having a “clearer” and “deeper” voices as compared to immature birds (Boeckle et al. 2018), immatures calling more often than adults (Sierro 2015), and adult females calling more often than adult males (Szipl et al. 2015). Still, individual-specific calling features remain a prominent factor in the analyses (c.f., Enggist-Dueblin and Pfister 2002), and can be picked up by listeners in playback experiments: i.e., in a habituation/dishabituation design, ravens discriminate unfamiliar birds, matched for age and sex, solely on the basis of their call structure (Boeckle et al. 2012). Moreover, when ravens could choose between two callers in a paired playback design, they were more attracted to the food calls of adult females than adult males, but only when these individuals were from the local community, i.e., the birds hardly approached the loudspeaker when food calls of unfamiliar individuals were played back (Szipl et al. 2015). These findings provide the first evidence that wild ravens take into account the familiarity of other non-breeders and treat local individuals differently from vagrants. Finally, recent observations showed that ravens were more likely to call when a social partner was in the vicinity but not yet at the foraging site and ceased calling as soon as the partner arrived (Sierro 2015). This raises the possibility that ravens may intend to recruit specific individuals, i.e., potential allies in the competition for food.

Competition for high-quality food such as carrion can be severe and often takes the form of aggression (Heinrich 1989). The chances of winning a conflict depends heavily on a raven’s age class (adults > immatures) and sex (males > females), but also on its bonding status and the quality of its social relationships, respectively: i.e., birds never engaging in affiliative interactions lose most fights, birds with increasingly strong relationships win increasingly more fights, and pair-bonded territorial birds win most fights (Gwinner 1964; Huber 1991). Note that these patterns emerge mainly as a result of passive social support (i.e., presence of an affiliate; Braun and Bugnyar 2012). Nevertheless, ravens may also get actively involved in others’ conflicts (Gwinner 1964), whereby they tend to support the aggressor (Loretto et al. 2012). However, if the victim is a close affiliate, they likely intervene on its behalf, even when the aggressor is higher in rank than themselves (Fraser and Bugnyar 2012). When being attacked, victims may utter defensive calls that function to appease the aggressor, but also alert the audience (Szipl et al. 2017). In a recent field study, victims were found to adjust their signaling to the audience composition: they increased calling when a close affiliate was in the audience but decreased calling when a close affiliate of the aggressor was in the audience (Szipl et al. 2017).

Taken together, these studies provide strong support for the assumption that raven non-breeder groups are more than anonymous crowds: i.e., individuals form differentiated relationships that are expressed in dominance and affiliation patterns. These studies also show that affiliative relationships are not restricted to future reproductive partners, as individuals form bonds with different partners (kin and non-kin) and often maintain more than one bond at a time (Braun and Bugnyar 2012). Still, raven affiliation networks remain relatively small in both captive and wild settings, hardly comprising more than two–five affiliates at a time (in the wild: Braun and Bugnyar 2012; in captivity: Kulahci et al. 2016). The quality of raven relationships moreover can be described by the components value, compatibility, and security (Fraser and Bugnyar 2010a), much in the same way as discussed for primate bonds (Fraser et al. 2008a). Specifically, the component value relates to the benefits associated with the relationship (e.g., allo-grooming, support in conflicts), compatibility to the extent of tolerance among partners, and security to the predictability of partners’ interactions and the relationship stability over time (Fraser et al. 2008a). Affiliates also show primate-like forms of post-conflict management such as reconciliation (Fraser and Bugnyar 2011) and bystander consolation to victims of aggression (Fraser and Bugnyar 2010b), indicating the importance of relationship repair and maintenance mechanisms (cf. Aureli and de Waal 2000).

## Social knowledge

Based on the importance of social relationships found in observational studies, we experimentally addressed the selective use of social relationships, and possible underlying mechanisms, in a series of cooperation studies with birds of our captive groups. As expected, ravens preferred to cooperate with their affiliates as compared to non-affiliates in experiments using the loose string paradigm (i.e., where two individuals have to simultaneously pull on two ends of a string to move a platform with a food reward inside reach; Asakawa-Haas et al. 2016). Success in this set-up was highly dependent on the tolerance for proximity between the cooperation partners, but also on the partners’ behavior in the previous trial, and ravens stopped cooperating when they had been cheated by their partner in respect to the reward distribution (i.e., the other got more than they themselves; Massen et al. 2015b).

In studies using the exchange paradigm (i.e., where an initial item is traded with a human experimenter for a better one), ravens stopped cooperating after witnessing another raven being rewarded for the same action with food of better quality or being rewarded for doing nothing (Wascher and Bugnyar 2013), indicating that they were sensitive to inequity in reward distribution and working effort. In a similar setting, ravens remembered fairly and unfairly behaving human experimenters in reciprocal interactions and avoided the unfair experimenter for at least 1 month after the initial cheat (Müller et al. 2017). Recent findings from Kabadayi and Osvath (2017) suggest that ravens might even be capable of future planning with regard to such bartering.

Finally, we used playback experiments to specifically test for the ravens’ knowledge about social relationships. Adult ravens that had left their captive group years ago instantly responded to hearing territory calls (“rab”) of former group members as compared to the same calls of unfamiliar birds (matched for age and sex; Boeckle and Bugnyar 2012). Among familiar callers, they even discriminated former affiliates from non-affiliates. These results clearly show that ravens are capable of remembering conspecifics on the basis of familiarity but also on their personal relationship valance (Boeckle and Bugnyar 2012).

Some of our field observations suggested that ravens may not only represent their own relationships (i.e., they can keep track with whom they affiliate or not, and to a certain extent, recall the history of past interactions with their different partners), but also take into account the relationships of others, so called third-party understanding. Inspired by the seminal work of Cheney and colleagues (Cheney et al. 1995; Bergman et al. 2003), we tested ravens’ third-party understanding in a playback experiment by simulating social interactions between group members of our captive groups (Massen et al. 2014a). Note that at the time of the study, we kept two groups of non-breeders in visual and auditory contact, allowing us to test birds about their knowledge of relationships between their group members and those of their neighbors. We played back dominance interactions that were either congruent with the existing dominance hierarchy (i.e., a dominant bird displacing a subordinate bird), or incongruent with the dominance hierarchy (i.e., a subordinate bird displacing a dominant bird), with the latter mimicking a dominance reversal. The tested ravens showed clear behavioral differences between these two conditions, confirming that in the incongruent condition the birds’ expectancy was violated. The fact that they also did so with the playback of the neighboring group suggests that they mentally represent the relationships of others, as in the case of those neighboring birds they could not use themselves as a reference point to infer the relationship of others (Massen et al. 2014a).

## “Politics”: Manipulating others’ relationships

Ravens, like many other animals (Massen et al. 2010), establish and maintain their relationships by seeking each other’s close proximity and preening each other (Fraser and Bugnyar 2010a). Occasionally a third raven intervenes in such affiliative behavior, making the others stop affiliating (Gwinner 1964). Observations at our field site suggest that these interventions are not random. Ravens that already have strong affiliative relationships specifically target those that are in the process of establishing such a relationship (Massen et al. 2014b). By doing so, they might prevent these birds from strengthening their relationship, and potentially, from rising in rank (Massen et al. 2014b). Note that ravens do not just intervene in any birds’ affiliative interactions, as they ignore the affiliative interactions of birds that have not yet established a relationship. They thus seem to monitor others’ interactions and take into account whether individuals exchange favors repeatedly and reciprocally (compare Hinde 1976). These observations raise the intriguing possibility that ravens not only represent others’ relationships but try to manipulate the formation of bonds, and consequently prevent future alliances (Massen et al. 2014b).

## Discussion

By reviewing more than 30 years of research, we reveal a differentiated picture of raven social life: (i) free-ranging non-breeding ravens may meet regularly, (ii) form social relationships, (iii) show a variety of behavioral maneuvers in competition for food and status, and (iv) rely on social knowledge for social-decision making. Playback experiments conducted on captive birds corroborate that: (v) ravens are sensitive to individual information in vocal communication and (vi) are capable of mentally representing their own and others’ relationships.

### Ravens’ social structure in comparison to other birds

Our findings clearly indicate that raven non-breeder groups can be more than “anonymous crowds,” which matches the observations of other bird species. Closely related corvids like rooks, *Corvus frugilegus*, and jackdaws, *Corvus monedula*, also form their first social bonds early in life, typically in the non-breeder state (von Bayern et al. 2007; Scheid et al. 2008), and adults may seek/keep social relationships in addition to their reproductive partner (in rooks: Boucherie et al. 2016, 2018), even though they differ in breeding style (ravens: territorial; jackdaws: semi-colonial; rooks: colonial). Taking a broader phylogenetical perspective, flocks of geese are structured by family units that actively support each other, and clans of related individuals that rest close to each other (Lorenz 1935, 1988; Lamprecht 1986; Scheiber et al. 2013). Foraging flocks of many parrots are characterized by overlapping home ranges, frequent exchange of flock members through fission-fusion events and reliance on social learning to accumulate foraging lore (Bradbury and Balsby 2016), resembling the foraging dynamics and movement patterns found in ravens. Members of parrot flocks may also show multiple individualized relationships, i.e., reproductive pairs and affiliative relationships among non-reproductive partners (e.g., Spoon et al. 2004; Hobson et al. 2014). Moreover, parrots show communicative interactions like short-term call matching to address specific group members (Wanker et al. 2005; Balsby et al. 2012) or vocal exchange to “negotiate” spacing (Bradbury and Balsby 2016).

Taken together, this confirms that monogamy does not prevent species from developing “complex” social structures, in and/or out breeding, i.e., with individuals relying on social information to make decisions and on the formation of individualized relationships to navigate their social environment. Hence, it shows that birds from different taxonomic groups rely on individualized relationships aside of their reproductive partnership and despite of a variability in group dynamics.

In several species mentioned above (i.e., corvids, geese, parrots), individuals are long-lived, and thus face a prolonged non-breeder period before the first reproductive attempts. This is not the case in all bird species. Although all bird species face such a transition period to adulthood and sexual maturity, we can expect its prevalence and duration to vary widely according to the species’ social system, life history traits (e.g., lifespan, age at first breeding attempt) and the patterns of natal dispersal (e.g., sex-bias, dispersal distances, and extent to which dispersal is constrained by ecological factors; Greenwood and Harvey 1982; Mulder 1995; Verhulst et al. 1997). Yet, the non-breeder state may also refer to a phase faced regularly throughout an adult bird’s life, i.e., the non-reproductive phase in a breeding cycle. Apart from ecological factors like seasonality in temperature and/or food availability, numerous species exhibit changes in social structure between breeding and non-breeding seasons. Taking corvid species as an example, irrespective of their mating and care systems (territorial, colonial, cooperatively breeding), the formation of “open” groups for foraging and roosting outside breeding seems to be the rule rather than the exception (Rowley 1973; Clayton and Emery 2007; Marzluff and Angell 2007; see also Holzhaider et al. 2011; St Clair et al. 2015). Seasonal variation in social dynamics and flocking behavior can also be observed in small bird species with a quick development and transition to breeding state, respectively (e.g., many songbirds defend territories in summer and flock in winter, Aplin et al. 2012; Silk et al. 2014).

A common feature of non-breeder aggregations across species and taxonomic groups seems to be a high variability in group dynamic and membership (Silk et al. 2014). In species forming individualized social relationships—like ravens—dealing with such an unpredictable social environment is assumed to be cognitively challenging, as individuals need to keep track of relationships in the absence of group members, infer relationships formed in their own absence, and possibly manage their social environment by selectively joining others (Aureli et al. 2008; see also Jolly 1966; Humphrey 1976 for the social intelligence hypothesis). Hence, we argue that the non-breeding period is key to fully understand social complexity and cognition in most avian species.

### Variation in the social environment and effect on social skills

A promising approach for an integrative view of avian social complexity may be to consider the types of challenges faced by birds in both the breeder and the non-breeder stage. Depending on the stage, individuals may face predominantly opposing or shared goals with other group members, which require different types of socio-cognitive skills. For instance, when young ravens join non-breeder groups, they are confronted with fierce competition for obtaining access and information about limited resources (Marzluff and Heinrich 1991; Bugnyar and Kotrschal 2002). In such a situation, it is highly advantageous to have “allies” in conflicts and the most reliable allies are affiliates (i.e., bonding partners; Silk 1982; Connor et al. 1992; Schino et al. 2007; Fraser and Bugnyar 2012). Hence, life in non-breeder groups should select for “Machiavellian” skills (Byrne and Whiten 1988), i.e., social knowledge and its tactical use (Heinrich 2011; Bugnyar and Massen 2017).

In contrast, when older ravens eventually settle for breeding as monogamous pairs, they face a highly cooperative situation, with reproductive partners sharing goals in respect to raising young, fending off predators and intruders (Lorenz 1937; Heinrich 1989). Such a situation may select for “cooperative”/Vygotkian skills (Moll and Tomasello 2007) like high levels of tolerance and coordination (Massen et al. 2015b), and possibly other-regard and empathy (Horn et al. 2016; but see Massen et al. 2015a; Lambert et al. 2017). Still, reproductive pairs may join non-breeders outside of breeding season, where they could rely on their Machiavellian skills again (e.g., to maximize their benefits in term of access to food). We thus likely find a mix of factors constituting social challenges, with some factors being more important than others at different life stages and/or seasons. Note that the relative importance of each factor/challenge may be different from species to species. Birds that exploit temporarily abundant food sources such as fruiting trees, as for example Pinyon jays (*Gymnorhinus cyanocephalus*), face little contest competition during foraging. Rather than outcompeting each other, they might benefit from collective behavior and instead of Machiavellian skills show highly coordinated behaviors among group members (Marzluff and Balda 1992; Bednekoff and Balda 1996; Duque et al. 2018).

### Ravens’ social cognition in comparison to other species

Our recent findings on ravens’ social knowledge fit well to those of “socially complex” mammals: dyadic *and* third-party knowledge has been experimentally demonstrated in nonhuman primates (Cheney and Seyfarth 1990, 2008; Silk 1999) and hyenas (Engh et al. 2005; Holekamp et al. 2007; see also in dolphins, Connor 2007; and in sea lions, Kastak and Schusterman 2002). Long-term memory for partners and relationships has been demonstrated in a variety of taxa, i.e., elephants (McComb et al. 2000, 2001), ungulates (Kendrick et al. 2001; Briefer et al. 2012), cetaceans (Bruck 2013), bats (Kerth et al. 2011), and carnivores (Pitcher et al. 2010). Moreover, how ravens use their social knowledge reflects many of the socio-cognitive maneuver reported for other animals, notably nonhuman primates. For instance, chimpanzees, *Pan troglodytes*, also alter their signaling depending on the audience: they exaggerate their screams in case the rank of one member of this audience at least, matches or surpasses the aggressor’s rank (Slocombe and Zuberbühler 2007). Chimpanzees also selectively choose cooperation partners in experimental settings (Melis et al. 2006), actively console their affiliates after a conflict (de Waal and van Roosmalen 1979; Fraser et al. 2008b), and intervene in others’ affiliative interactions, possibly to prevent them from forming bonds (de Waal 1982; see also Mielke et al. 2017). Selectivity in cooperation has also been demonstrated in monkeys (e.g., in Barabary macaques, Molesti and Majolo 2016; in capuchin monkey, de Waal and Davis 2003), other corvids (e.g., in rooks, Seed et al. 2008; Scheid and Noë 2010), elephants (Plotnik et al. 2011), wolves (Marshall-Pescini et al. 2017), and parrots (Schwing et al. 2016). Post-conflict consolation have been described for some primates (review in Fraser et al. 2009; see also Palagi et al. 2004; Cordoni et al. 2006), canids (Palagi and Cordoni 2009), and other corvids (Seed et al. 2007; Logan et al. 2013) and “political” interventions in others’ affiliation and agonistic interactions have been reported from a few species such as wolves, primates, and horses (Ward et al. 2009; Krueger et al. 2015; Mielke et al. 2017).

Hence, problems associated with dealing with social relationships are apparently solved in a similar way across distantly related species. What is yet unclear is whether the behavioral similarities between different taxa are also based on the same cognitive mechanisms. The recent findings in ravens hint towards similarities on the behavioral and cognitive level, supporting the idea of convergent evolution of socio-cognitive skills between birds and mammals (Clayton and Emery 2004), despite radical different brain structures (Güntürkün and Bugnyar 2016) and, importantly, despite different social systems.

## Conclusion

As in mammals, a substantial part of avian social complexity lies in the variability and unpredictability of the social environment in which birds navigate (e.g., formation of open groups characterized by high degrees of fission-fusion dynamics). Furthermore, we now have good evidences that a monogamous mating system does not prevent avian societies from becoming “complex” and cognitively challenging in terms of social relationships. On the contrary, the monogamous mating system of many bird species makes them prone to use bonding partners as allies in conflicts and for gaining status. In addition, such affiliative relationships may go beyond reproductive partners and extend to kin and “friends” in the non-breeding period, in which individuals also experience high dynamics in group formation (see Fig. 1). Taking an individual’s perspective on the challenges faced in different social settings within a species may be a promising approach to investigate ‘social complexity’ of birds, as it allows an integrative view across life history stages and social contexts. Ultimately, the combination of this individual level perspective with the large-scale perspective on social systems should allow us to better apprehend the diversity of evolutionary routes that might have led to sociality and social complexity across species and taxa.
